# Industrial camera model positioned on an effector for automated tool center point calibration

**DOI:** 10.1038/s41598-023-51011-5

**Published:** 2024-01-03

**Authors:** Jaromir Konecny, Petr Beremlijski, Michaela Bailova, Zdenek Machacek, Jiri Koziorek, Michal Prauzek

**Affiliations:** VSB – Technical University of Ostrava, 17. listopadu 2172/15, Ostrava, 708 00 Czech Republic

**Keywords:** Engineering, Information theory and computation

## Abstract

The study presents a novel, full model of an industrial camera suitable for robotic manipulator tool center point (TCP) calibration. The authors propose a new solution which employs a full camera model positioned on the effector of an industrial robotic arm. The proposed full camera model simulates the capture of a calibration pattern for use in automated TCP calibration. The study describes an experimental test robot stand for producing a reference data set, a full camera model, the parameters of a generally known camera obscura model, and a comparison of proposed solution with the camera obscura model. The results are discussed in the context of an innovative approach which features a full camera model to assist the TCP calibration process. The results showed that the full camera model produced greater accuracy, a significant benefit not provided by other state-of-the-art methods. In several cases, the absolute error produced was up to seven times lower than with the state-of-the-art camera obscura model. The error for small rotation (max. of 5$^\circ$) and small translation (max. of 20 mm) was 3.65 pixels. The results also highlighted the applicability of the proposed solution in real-life industrial processes.

## Introduction

One of the pillars of Industry 4.0 is the widespread deployment of industrial robots in manufacturing. Mechanical assembly processes are key manufacturing stages, and the use of robots in these processes directly affects product quality by improving production efficiency and assembly performance^1^.

Industrial robotic manipulators have excellent repeatability and accuracy for precise positioning, but because they are used under intensive operating conditions, they must be repeatedly calibrated during the manufacturing process^2^. During calibration, the position and orientation of the tool center point (TCP) of a robot arm should be corrected with a highly accurate tracking device^3^. Numerous methods for calibration procedures have been published. Khaled et al.^4^ proposed an active disturbance rejection control scheme which calibrated path accuracy in real time. Fares et al.^5^ applied a sphere fitting algorithm to improve four point calibration accuracy.

The primary objective of this article is to develop a camera model capable of simulating the positions of anchor points within a calibration pattern as captured from various camera positions and orientations. This model can subsequently be utilized to resolve the inverse problem: determining the camera’s position and orientation based on the locations of the anchor points in the captured image. The accurate determination of the camera’s position can then aid in calibrating the TCP of a robotic arm.

**Figure 1:**
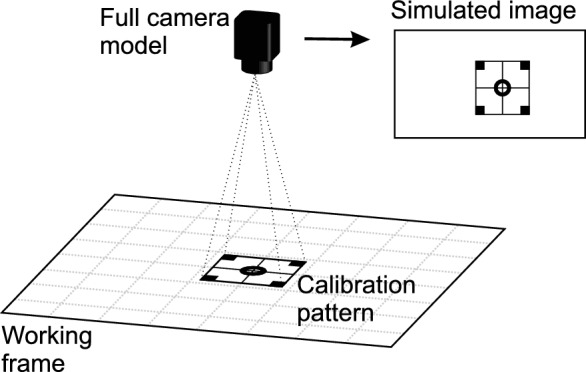
Camera model capturing a calibration pattern to estimate the position and orientation of the calibration pattern.

This article presents a full model of an optical camera used in combination with the calibration procedure (Fig. 1). The proposed camera model is able to capture an image of a calibration pattern from a certain camera position. The calibration pattern has defined corners and a center point positioned according to the coordinate system of the working plane. The image depicts the calibration pattern point positions as equivalent as possible to an image captured by a real-life camera . The proposed camera model can be easily adapted to an effector for calibration of the robot TCP.

The study contributes the following:

1. Design of a full camera model.
2. Comparison with a previously published camera obscura model.
3. Application of a full camera model in calibrating a robotic manipulator’s TCP.

Calibrating robotic manipulators is an important aspect of any industrial robot deployment. The aim of TCP calibration is to determine the relationship between the TCP frame and working plane^6^. Calibration is performed using the camera in the robot’s end-effector. The camera captures an image and the calibration point positions are then calculated. However, the calibration procedure requires an inverse problem solver, where the TCP is actually calculated from the calibration points captured on the image. An inverse problem solver is obtained through an iteration process which determines the combination of input values (camera position and orientation), resulting in the calibration point positions in the modelled image, which is as close as possible to the real camera image captured by the robotic tool. A gradient method or difference evolution, for example, can be used as an optimization solver^7,8^.

The article is organized as follows: Section "Introduction" introduces the scientific challenge and novelty of the current study; Section "Related studies" summarizes the state-of-the-art; Section "Mathematical model" describes the camera and the full camera model; Section "Experiment" outlines the experiment, reference data set and evaluation criteria; Section "Results" reports the results of the experiment from absolute and relative error perspectives; Section "Discussion" discusses and evaluates the results in the context of the study’s novel contribution; Section "Conclusion" concludes the article and outlines potential future work.

## Related studies

Precision robot guidance is a critical parameter in robotic manipulators. Robotic manipulators have inaccurate positioning yet excellent repeatability, which means that any error or inaccuracy in movement is also repeated^9^. The scientific literature contains many methods for improving precision during robot movement. Table 1 provides a summary of state-of-the-art studies which propose solutions for robot movement and detection of position, robot coordination sensors and camera obscura modelling methods.

**Table 1:** Related state-of-the-art studies.

Topic	Content
Sensors for robot coordination	
Camera	Detection of holes and calculation of position in 3D space^10^. Inflatable robotic arm with camera system for highly accurate positioning^11^.
3D sensors	Combination of depth sensor and camera data^12^. RGBD camera used as a depth sensor for spatial and object recognition^13^.
Combinations and other methods	Combination of a global laser and local scanning system^14^. Visual reconstruction using a planar laser, two cameras and 3D orientation board^15^. Planar-laser-guided solution with a unique reference point and sensor module mounted with a photoelectric position sensitive detector^16^. Visual-tactile sensor^17^. Optical fibers^18^.
Robot orientation and detection of position	
Optimization methods	Optimization of 3D manipulator position by combining a camera and robot model algorithms^19^. Optimization of robotic manipulator movements for an inspection system^20^.
Machine learning algorithms	Detection system used by decoration robots for wall bulge endpoints regression and classification according to orientation^21^. Self-supervised representation learning networks for accurate spatial guidance^22^. Orientation and operation of a robotic system with variable products by processing 3D scanner data^23^.
Image processing methods	Vision-based guidance of a robotic arm for object handling operations^24^. Automated object sorting using a robotic arm with a Kinect sensor^25^. Dynamic proportional-fuzzy grip control for a robotic arm using a two-dimensional vision sensing method^26^.
Camera obscura modelling	
Improvement in model accuracy	Eye camera obscura model augmented with geometric algebra to characterize eye position and rotation axes^27^. Camera obscura calibration using planar template images for focal length changes^28^. Combination of a camera obscura model and non-metric and self-calibration methods^29^. Vision-based adaptive control algorithm for positioning with a camera obscura^30^.
Practical implementation	Simplified camera modeling in overhead line navigation^31^. Creation of a camera for solar pointing to a precision of 0.01$^{\circ }$^32^. Detection of the distance and dimensions of a tracked object using an algorithm in combination with a camera obscura^33^.
Calibration and positioning	Calibration of industrial robots with an absolute position tracking system^34^. Self-calibrating camera algorithm for an active vision system which detects radial distortion^35^. Robot positioning using camera-apace manipulation with a linear camera model^36^.

Industrial producers devise calibration procedures generally based on manual positioning of various types of spike. To achieve greater precision than manual calibration or to calibrate robotic arms automatically, additional sensors must be used^37^. One option is to use a camera to observe manufactured pieces and discover fitting patterns with subsequent image processing and then calibrate the robot’s coordination frame according to these patterns^10^. A camera can be also used to observe the robotic arm itself and provide visual feedback on its position^11^.

Another option is to use a 3D sensor, which, in contrast to a 2D camera, provides additional depth information. RGBD cameras are suitable types^12^ that record depth information for each RGB pixel. An example of RGBD camera application is the detection of cardboard box position without any markings^13^.

For large areas requiring precise positioning, the combination of a global laser and local scanning system can be used^14^. Chen et. al.^15^ introduced visual reconstructions using a planar laser, two cameras and a 3D orientation board to verify the accuracy of an active vision system’s measurement fields and calibration. Other methods involve the use of visual-tactile sensors^17^ or optical fibers^18^.

Accuracy in robot guidance can be improved with various mathematical approaches. Lei et al. applied Levenberg-Marquardt optimization for 3D pose estimation^19^. The robotic arm itself can be also used for inspection points. Tabu search can find the optimal base position, and the Lin-Kernighan method can optimize the order of inspection points^20^. Machine learning methods also provide promising solutions for positioning robotic arms. Eldosoky et al. applied a machine learning algorithm in a detection system used by in decoration robots for wall bulge endpoints regression and classification according to its orientation^21^. Choudhary et. al. introduced self-supervised representation learning networks for accurate spatial guidance^22^. Gouveia et. al. described a smart robotic system which processed spatial data obtained from a 3D scanner and was capable of performing an autonomous pick-and-place task of injected moulded parts from one conveyor belt to another^23^. Image processing can also enable automated calibration of robotic arms^24^. Specialized RGBD cameras allow robots to “see” objects like a human^25^. Image processing also solves the challenge of grip control in handling moving objects^26^.

Image processing methods often employ cameras such as a pinhole cameras and are widely used in industrial applications^27^. Table 1 provides a summary of improvements, practical implementations and calibration and positioning methods for camera obscura models. Camera obscura models assume that the camera’s parameters are correct and have a projection error of almost zero. Because all the camera parameters are known, any computed results are considered true. However, the camera obscura model does not accurately represent camera behavior if an autofocus device is used^28^. Lens distortion is not a problem with camera obscura models^29^. Image projection errors can be minimized with backstepping-based approach which synthesizes the Lipschitz condition and natural saturation of the inverse tangent function^30^.

Camera obscura models have many practical applications, such as detecting and avoiding obstacles^31^, precise solar pointing^32^ or determining the distance and dimensions of a tracked object^33^. Camera obscura models can be also used in the calibration process for industrial robots^34^. Liu et al. devised a self-calibration camera algorithm for an active vision system which detects radial distortion^35^. Rendón et al. presented a method for positioning robots using camera-space manipulation with a linear camera model^36^.

## Mathematical model

This section presents the optical equations for a thin lens camera and a camera obscura (pinhole camera) and two mathematical models based on these equations. A complicated mathematical model describes the thin lens camera, whereas a simplified state-of-the-art model characterises the pinhole camera.

### Optical equations

To create the mathematical model of a camera with a thin lens, we used the optical equation

1 $$\begin{aligned} \frac{h'}{h} = \frac{f}{a-f}, \end{aligned}$$

where the focal length is *f*, the symbol *a* denotes the distance of an object from the lens, *h* is the object’s height, and $h'$ is the image height.

A pinhole camera is a simple type of an optical device and predecessor to cameras with a thin lens. A camera obscura is a box with a hole in one of its walls. Light from outside passes through the hole hits and projects an image onto the opposite wall.

The mathematical model of a camera obscura is described by the equation

2 $$\begin{aligned} \frac{h'}{h} = \frac{a'}{a}, \end{aligned}$$

where $a'$ is the focal distance. This simple camera model is popularly used in many applications for its simplicity and ease of implementation.

### Model implementation

In this section, we derive a model with a thin lens. This type of model is more complex than a camera obscura, but it takes advantage of the properties of modern cameras. Equation (1) was used to create the mathematical model.

Manufacturers do not usually provide a precise position of the lens, therefore , a displacement *L* is described in the mathematical model of the full camera. A camera lens can thus be positioned at a different point to the TCP since both points lie on the camera axis *o* (see Fig. 3). The distance between these points is denoted *L*. We thus obtain the Equation (1)

3 $$\begin{aligned} \frac{h'}{h} = \frac{f}{a+L-f}. \end{aligned}$$

**Figure 2:**
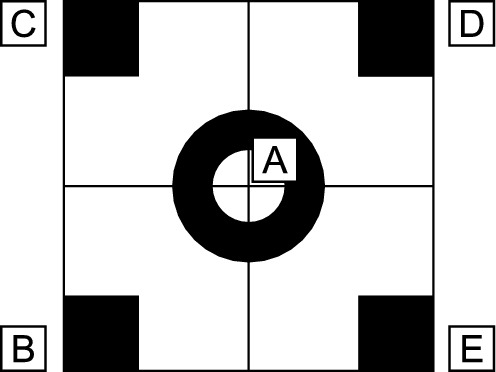
Geometric pattern displayed by the camera.

This optical equation is applied to a calibration square of prescribed geometric shape (Fig. 2).

In the mathematical model, the projected points of the calibration square (located in Euclidean space $\mathbb {R}^3$) must correspond to points on the camera chip (in the plane $\mathbb {R}^2$). The mapping is denoted *F* and must satisfy (3).

**Figure 3:**
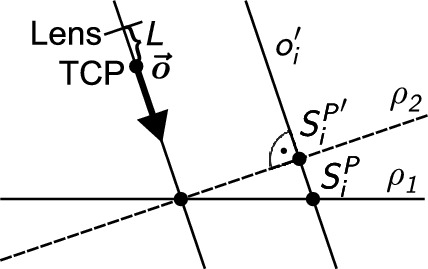
Orthogonal projection of a point on the calibration square onto a plane perpendicular to the camera axis – 2D view.

The calibration square contains five points described by

4 $$\begin{aligned} P=[A,B,C,D,E]=[S_1^P,S_2^P,S_3^P,S_4^P,S_5^P], \end{aligned}$$

where *A*, *B*, *C*, *D* a *E* refer to the points indicated in the diagram (Fig. 2). Points $S_i^P$ belong to the plane $\rho _1$ ($\rho _1: z=0$ in the case described in this paper). To correctly apply the optical Eq. (3), the calibration square must be projected orthogonally onto the plane $\rho _2$ perpendicular to the camera axis *o* (defined by the rotation of the camera) and $\rho _1$. Figure 3 illustrates the orthogonal projection of a calibration square point onto a perpendicular plane.

The camera lens is positioned at a point shifted from the TCP by the distance *L* in the direction of the *o* axis and focused on the point $Q \in \rho _1 \cap \rho _2$.

To define the plane $\rho _2$, its normal vector given by the camera axis *o* is required. It is assumed that the initial rotation of the camera is given by the vector $\vec {v}$. For example, if the camera in its initial position is rotated according to a *z*-axis only, then

5 $$\begin{aligned} \vec {v}=\left[ \begin{array}{c} 0\\ 0\\ -1 \end{array}\right] . \end{aligned}$$

If the angles $\alpha$, $\beta$ a $\gamma$, describing the rotations with respect to the *x*, *y* and *z* axes are known, then vector *o* can be computed using the rotation matrix

6 $$\begin{aligned} R=R_x(\alpha )R_y(\beta )R_z(\gamma ), \end{aligned}$$

where

7 $$\begin{aligned} R_x(\alpha )=\left[ \begin{array}{ccc} 1 &{} 0 &{} 0 \\ 0 &{} \cos \alpha &{} - \sin \alpha \\ 0 &{} \sin \alpha &{} \cos \alpha \\ \end{array} \right] ,~R_y(\beta ) = \left[ \begin{array}{ccc} \cos \beta &{} 0 &{} \sin \beta \\ 0 &{} 1 &{} 0 \\ -\sin \beta &{} 0 &{} \cos \beta \\ \end{array} \right] ,~R_z(\gamma ) = \left[ \begin{array}{ccc} \cos \gamma &{} -\sin \gamma &{} 0 \\ \sin \gamma &{} \cos \gamma &{} 0 \\ 0 &{} 0 &{} 1 \\ \end{array} \right] . \end{aligned}$$

For the direction vector *o*, we have

8 $$\begin{aligned} \vec {o}=R \cdot \vec {v}, \end{aligned}$$

where $\vec {v}=[v_1,v_2,v_3]^T$. The general equation of the plane $\rho _2$ can be derived from the conditions

9 $$\begin{aligned} \rho _2 \bot o \, \wedge \, Q \in \rho _2,\mathrm {~where~}Q \in o \cap \rho _1. \end{aligned}$$

**Figure 4:**
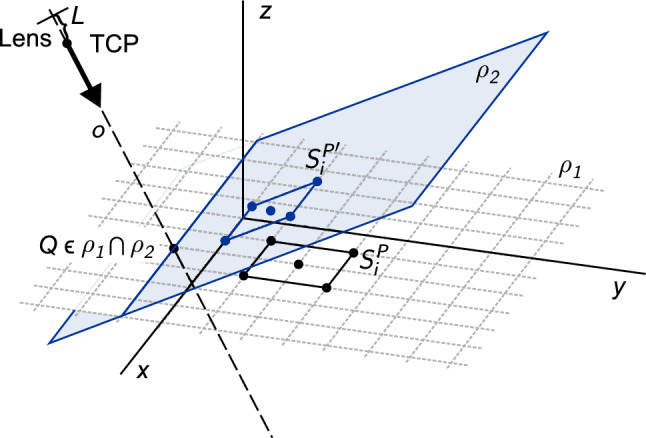
Orthogonal projection of a geometric pattern onto a plane perpendicular to the camera axis.

Figure 4 illustrates the projection of a geometric pattern onto a plane perpendicular to the camera axis.

Orthogonal projection of $S_i^P \in \rho _1$ onto a point $S_i^{P'} \in \rho _2$ is possible. Using analytical geometry, the orthogonal projection $S_i^{P'}$ can be written as

10 $$\begin{aligned} S_i^{P'}= & {} S_i^P+\frac{\vec {o}^TQ-\vec {o}^TS_i^P}{\Vert \vec {o}\Vert ^2}\vec {o} =S_i^P+\frac{\vec {v}^TR^T_z(\gamma )R^T_y(\beta )R^T_x(\alpha )Q-\vec {v}^TR^T_z(\gamma )R^T_y(\beta )R^T_x(\alpha )S_i^P}{\Vert \vec {o}\Vert ^2}\cdot R^T_z(\gamma )R^T_y(\beta )R^T_x(\alpha )\vec {v} \nonumber \\= & {} S_i^P+\frac{\vec {u}^T(Q-S_i^P)\vec {u}}{\Vert \vec {o}\Vert ^2}\vec {u}, \end{aligned}$$

where

11 $$\begin{aligned} \vec {u}=R(\alpha ,\beta ,\gamma ) \vec {v}. \end{aligned}$$

**Figure 5:**
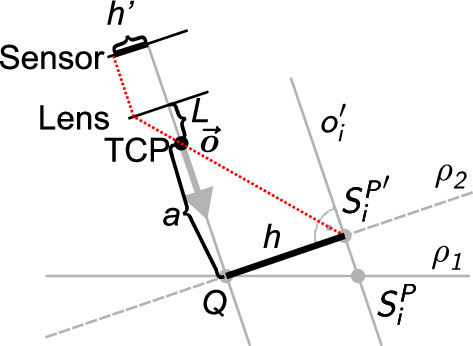
Orthogonal projection of a geometric pattern onto a plane perpendicular to the camera axis and its display on the camera’s graphics chip.

In optical Eq. (3), let $a: \mathbb {R}^3 \rightarrow \mathbb {R}$ denote the distance between the TCP with coordinates [*x*, *y*, *z*] and the calibration square (Fig. 5).

To apply (3), *a*, *h* and $h'$ must be calculated for each point of the calibration square. The value of *a* for the position [*x*, *y*, *z*] can be computed from

12 $$\begin{aligned} a(x,y,z)=\left\| Q-[x,y,z]\right\| . \end{aligned}$$

Again, we have the condition

13 $$\begin{aligned} Q \in o \cap \rho _1 \end{aligned}$$

to express the coordinates of *Q*; substituting into (12), we obtain an expression for *a* with respect to the camera’s position [*x*, *y*, *z*] and its rotation described by matrix *R*:

14 $$\begin{aligned} a(x,y,z)=\left\| \left[ -\frac{R_1\vec {v}}{R_3\vec {v}}z,-\frac{R_2\vec {v}}{R_3\vec {v}}z,-z\right] \right\| , \end{aligned}$$

where $R_i$, $i \in \{1,2,3\}$ denotes an *i*-th row of matrix *R*.

The value of the object’s height *h* for each point $S_i^{P'}$ is computed from

15 $$\begin{aligned} h_i=\Vert S_i^{P'}-Q\Vert . \end{aligned}$$

Substituting the value of (15) into (3), we have

16 $$\begin{aligned} h'_i=\frac{f}{a+L-f}h_i=\frac{f}{a+L-f}\Vert S_i^{P'}-Q\Vert . \end{aligned}$$

It is worth mentioning that $h'_i$ is the height of the image displayed by the camera chip and therefore belongs to $\mathbb {R}^2$; thus

17 $$\begin{aligned} S_i^{P''}=Q''+\frac{(S_i^{P'}-Q)}{\Vert S_i^{P'}-Q\Vert }h'_i \in \mathbb {R}^2, \end{aligned}$$

where $Q'' \in \mathbb {R}^2$ is the center of the camera chip’s display plane (here, $Q''=[0,0]$) and $S_i^{P''}$ is an image of $S_i^{P'}$ in the camera chip).

Finally, we obtain the function $F:\mathbb {R}^6 \rightarrow \mathbb {R}^{10}$, where

18 $$\begin{aligned} F(x,y,z,\alpha ,\beta ,\gamma )=[S_1^{P''},\dots ,S_5^{P''}]^T=\left[ Q''+\frac{(S_1^{P'}-Q)}{\Vert S_1^{P'}-Q\Vert }h'_1, \dots , Q''+\frac{(S_5^{P'}-Q)}{\Vert S_5^{P'}-Q\Vert }h'_5\right] ^T. \end{aligned}$$

The vectors

19 $$\begin{aligned} \frac{(S_i^{P'}-Q)}{\Vert S_i^{P'}-Q\Vert }=[k_1^i,k_2^i] \end{aligned}$$

can be obtained using the orthogonal direction vectors of the plane which contains the camera chip’s display plane.

It is important to recognize that when utilizing a static camera, a linear relationship exists between the size of the object and the size of its image. Conversely, in our proposed model featuring a non-stationary camera, the function *F* – which characterizes the image output on the camera sensor in relation to the camera’s position and orientation – exhibits a nonlinear nature. This nonlinearity arises because the angles defining the camera’s orientation are not sufficiently small to permit linearization of the model.

### Implementation of reference model

The current study discusses the use of a camera obscura as a reference model. A camera obscura is typically defined as a linear system described by matrix multiplication^38,39^. However, this type of model is used for static cameras and changing images only. The camera investigated in the current study is not static, therefore we derive camera obscura model from general optical equations.

As with the full camera model, the distance between the TCP and the lens is denoted *L* for the camera obscura, giving the equation

20 $$\begin{aligned} \frac{h'}{h} = \frac{a'}{a+L}. \end{aligned}$$

For the camera obscura model, we also need to derive a vector function $F:\mathbb {R}^6 \rightarrow \mathbb {R}^{10}$ to describe the relationship between the calibration square’s image and the camera’s position and rotation. To formulate the mathematical model of the camera obscura, it is again necessary to describe the projection of the calibration square onto the camera chip. We obtain the camera obscura equations as follows:

1. Determine the plane $\rho _2$ perpendicular to the camera axis and which intersects the calibration pattern plane on the camera axis (Fig. 4 and Eq. (9)).
2. Obtain an orthogonal projection of the calibration square onto the plane $\rho _2$ (Fig. 4 and equation (10)).
3. Calculate the distance from the object *a* (Eqs. (12, 13, 14)).
4. Calculate the image height $h'$ from the camera obscura optical Eqs. (15) and (20). This results in:
  21 $$\begin{aligned} h'_i=\frac{a'}{a+L}h_i=\frac{a'}{a+L}\Vert S_i^{P'}-Q\Vert , \end{aligned}$$
  where $a'$ is constant.
5. Obtain the mathematical model of the camera obscura by substituting Equation (21) into (18).

Deriving the mathematical model of the camera obscura, we obtain the function *F* from Equation (18), although $h'$ is obtained from Eq. (21). Function *F* is a common derivation, therefore it is not presented here in detail.

## Experiment

The proposed model was set up in Matlab, and its functionality and accuracy were tested with experimental measurements and a comparison to previously published camera obscura models.

### Experimental setup

**Figure 6:**
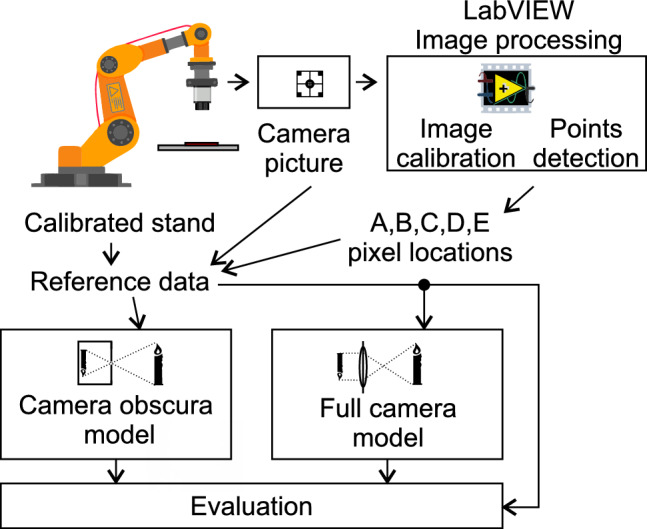
Schematic drawing of the experimental setup.

To evaluate the functionality of the proposed camera model, we designed the experimental setup illustrated in the scheme in Fig. 6.

A calibrated robotic arm and camera were first set to a known position and an image of the calibration pattern on the work plane was captured. The image was processed in LabVIEW to determine the positions of the pattern’s anchor points. The image processing procedure encompasses image calibration to eliminate radial and tangential distortion, along with the detection of anchor points. Radial and tangential distortions are removed through camera calibration, which is determined prior to capturing the reference data set in accordance with the national instrument calibration procedure. The anchor point (A–E) *x* and *y* coordinates, calibrated robotic arm position (and orientation) and camera image formed a reference data set.

The reference data set was then used as input for other camera models. In the experiment, we tested and evaluated a commonly used, state-of-the-art camera obscura model and a mathematical model for a full camera and then compared the results to determine any difference in behaviour. The mathematical models used the robotic arm’s reference position and estimated the coordinates of anchor points A–E. These coordinates were then compared to the real measurements from the camera in the evaluation block.

### Reference data

For evaluation purposes, we assembled a test robot stand and experimentally measured a reference data set.

**Figure 7:**
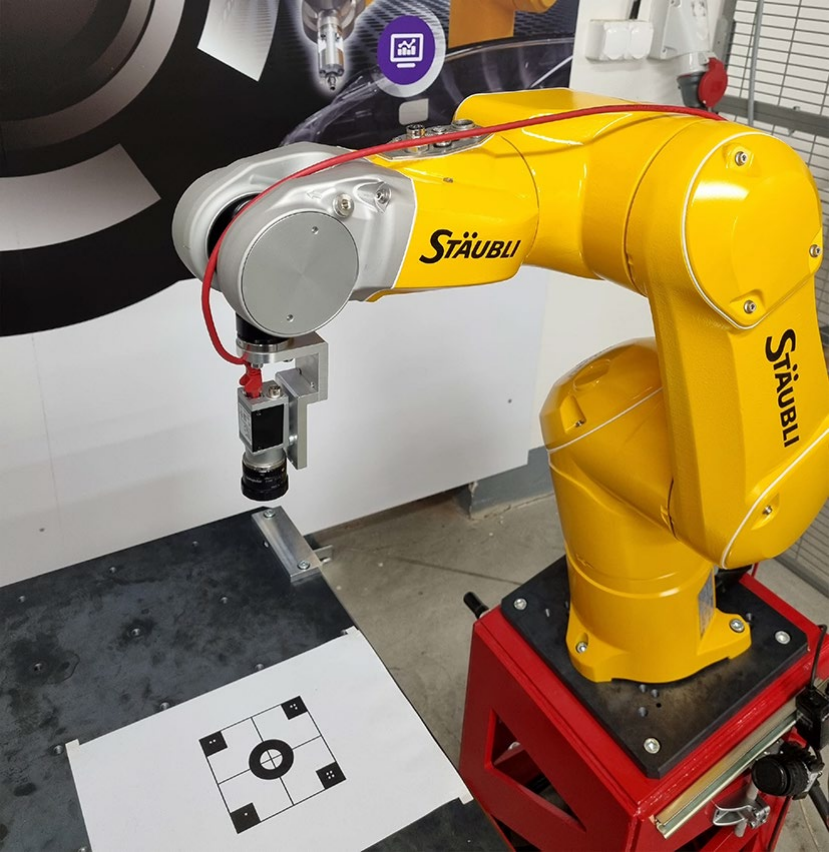
Photo of test robot stand with fixed camera on the effector and printed calibration pattern.

Figure 7 shows a photo of the test robot stand. This experimental setup consisted of a Staubli TX2-60 industrial 6-axis robotic manipulator (controller CS9 s8.12.2-Cs9_BS2561)^40^ and a Basler acA2500-14gm camera^41^ with a Computar M0814-MP2 2/3” 8 mm f1.4 lens^42^, a work table and additional hardware to permit manual calibration.

The calibration procedure for the test robot stand consisted of two consecutive steps: rough calibration and fine calibration. Rough calibration involved a three point TCP calibration method using spikes, designed in cooperation with engineers from Staubli. Fine calibration involved precise positioning of the pattern at the center of the camera image and setting the camera axis perpendicular to the calibration pattern plane. The pattern was then replaced with a mirror and the camera aligned so that its reflection was in the exact center of the lens and middle of the image.

The accuracy of robot positioning was estimated at ±0.1 mm. An error of 1 pixel in detecting the anchor point and an error of approximately 0.1 mm related to the camera’s resolution and calibration pattern size were also accounted for. The total error was estimated as a Euclidean norm at 0.14 mm.

**Figure 8:**
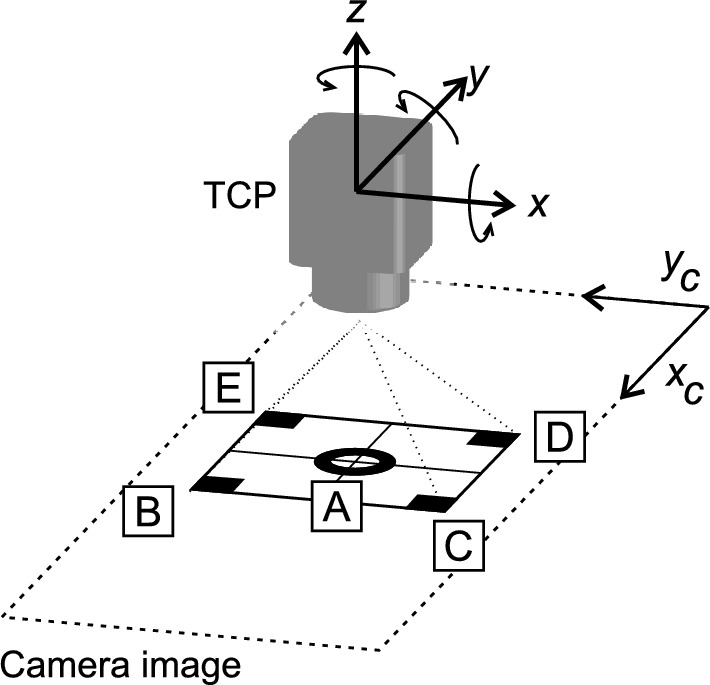
Coordinate system definition: TCP coordinate system and camera image coordinating system.

The reference data set consisted of 100 positions and TCP orientations plus a base position. The camera’s initial placement was 300 mm above the center of the geometric figure. Figure 8 illustrates the TCP and camera coordinate systems. The robotic arm was progressively moved along the *x*, *y* and *z* axes, − 50 mm to 50 mm on the *y* and *z* axes and − 20 mm to 20 mm on the *x* axis. Movement along the *x* axis was tested on a limited range because the camera’s field of view in the horizontal direction was limited. The data set also contained various orientations for the base point from − 20$^\circ$ to 20$^\circ$. The range of rotation on the *y* axis was limited to -20$^\circ$ to 10$^\circ$ due to possible collision with robot’s fourth axis. The reference data set also contained several additional measurements of combinations of rotation and translation of the TCP.

Table 2 lists the parameter values used in the experiment. The parameters, including focal length (*f*) and camera resolution, were specified by the manufacturer of the camera. Additionally, the calibration square was precisely fabricated by an industrial partner. The sole parameter determined through experimental methods was the distance (*L*). A detailed sensitivity analysis of the parameter *L* is discussed in Section "Discussion".

**Table 2:** Camera parameters used in the experiment.

Parameter	Value
Focal length *f*	8 (mm)
Distance *L*	9.5 (mm)
Calibration square size	$100 \times 100$ (mm)
Camera resolution	$2592 \times 1944$ (px)

### Evaluation criteria

The study applied three evaluation criteria to compare the results from the mathematical models of the full camera and camera obscura. The first criteria is the difference in distance between the anchor point positions obtained from each camera model; these figures are independent of the calibration pattern size and camera properties. The difference was calculated from the average Euclidean distance between the real anchor points positions and the mathematical model anchor point positions:

22 $$\begin{aligned} \mathrm{Abs.\ Err.\ (px)} = \frac{1}{5} \sum \limits _{A,B,C,D,E} \sqrt{(A_{\textrm{ref} x} - A_{x})^2 + (A_{\textrm{ref} y} - A_{y})^2}, \end{aligned}$$

where *A*–*E* are anchor points, ${\textrm{ref}}$ are reference anchor points, and Abs. Err is the absolute error in pixels.

The second criteria is the absolute error in millimeters produced by the test robot stand with the specific pattern size and camera model. The absolute error in millimeters directly relates to the calibration pattern size. The calibration pattern was printed on paper with a laser printer, the edge of the calibration pattern being 100 mm in length. To analyze the absolute error in millimeters, a projective transformation was applied. The absolute error in millimeters was calculated from

23 $$\begin{aligned} \mathrm{Abs.\ Err. (mm)} = \frac{1}{5} \sum \limits _{A,B,C,D,E} \sqrt{\left[ \textbf{T}(A_{\textrm{ref} x}) - \textbf{T}(A_{x})\right] ^2 + \left[ \textbf{T}(A_{\textrm{ref} y}) - \textbf{T}(A_{y})\right] ^2}, \end{aligned}$$

where $\textbf{T}$ is a projective transformation which transfers the image plane in pixels to the calibration pattern plane in millimeters.

The third and final evaluation criteria is the relative error, which relates to the diagonal distance of the calibration pattern; this figure is independent of the pattern size and camera model. The relative error was calculated from

24 $$\begin{aligned} \mathrm{Rel.\ Err.} = \frac{1}{5} \sum \limits _{A,B,C,D,E} \frac{ \sqrt{(A_{\textrm{ref} x} - A_{x})^2 + (A_{\textrm{ref} y} - A_{y})^2} }{\sqrt{(C_{x} - E_{x})^2 + (C_{y} - E_{y})^2}}. \end{aligned}$$

## Results

This section discusses the results of the comparison between a commonly used camera obscura model and the proposed full camera model. Both models are compared to a reference real camera solution.

**Figure 9:**
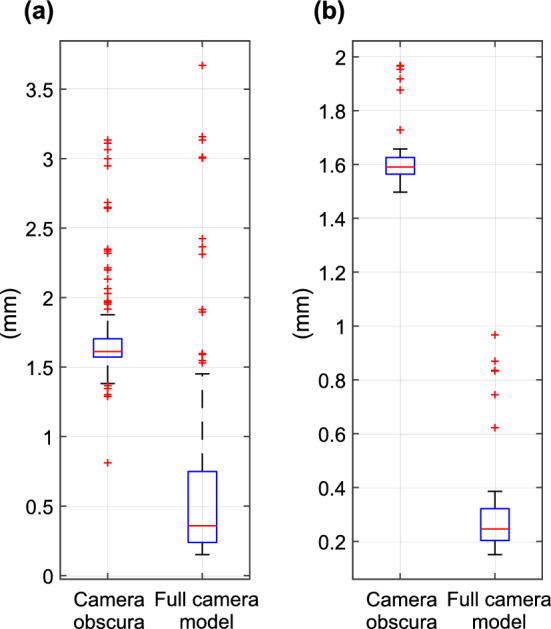
Box plots of absolute errors in millimeters : (**a**) entire data set; (**b**) subset containing translations not greater than 20 mm and rotations not greater than 5$^\circ$.

Figure 9 compares the results for the models’ overall accuracies. The box plots indicate absolute errors in millimeters . Figure 9A shows a comparison of the absolute errors in millimeters for the entire dataset, which includes the reference data and various translations, rotations and combinations of translation and rotation. Figure 9A indicates a significantly lower absolute error from the full camera model than the camera obscura model. For calibration of the robotic manipulator, we assume that the operator manually places the effector in the approximate desired position. This position is assumed to be no greater than the translation distance (20 mm) or rotation (5$^\circ$) on each axis. Figure 9B plots a comparison of the camera obscura model and full camera model for small translations and rotations. Significantly, the median absolute error from the full camera model is approximately seven times lower than absolute error from camera obscura model.

**Figure 10:**
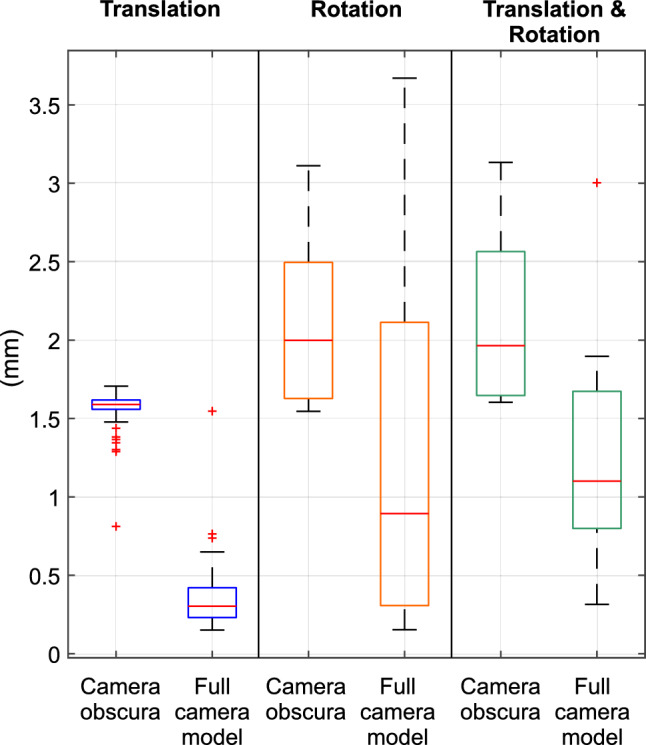
Box plots of absolute errors in millimeters : Translation – subset contains translations only; Rotation – subset contains rotations only; Translation & Rotation – subset contains a combination of translations and rotations.

The box plots in Fig. 10 indicate the absolute errors in millimeters for each subset. The first two box plots (Translation) indicate the absolute error in millimeters for robotic arm translation only. The difference between the absolute errors for translation was significant, the full camera model producing an error six times lower than the camera obscura model. The two box plots in the middle indicate the absolute errors for TCP rotation. Improvement in the full camera model was not so significant as in the case of translation, but it is still lower than the error produced by the camera obscura model. Similar results were obtained for the combination of translation and rotation (box plots Translation & Rotation), showing an absolute error 1.82 times lower in the full camera model.

Table 3 summarizes the results for the camera obscura and full camera models, indicating averages for various input data sets. The first row states the results for the entire data set. Comparing the results, that full camera model produced absolute and relative errors approximately 2.65 times lower than the camera obscura model. The second row indicates the results for small rotation and translation. As mentioned above, we assumed that an operator manually navigates the robotic arm near the desired TCP during calibration process. For small rotation and translation, the full camera model produced errors 5 times lower than the camera obscura.

**Table 3:** Summary of results for the camera obscura and full camera models, indicating averages for each input data subset.

Case	Abs. Err. (px)		Abs. Err. (mm)		Rel. Err. (%)	
	CO	FCM	CO	FCM	CO	FCM
Overall	21.43	8.06	1.76	0.68	1.30	0.49
Small rot. and trans.	19.64	3.65	1.63	0.32	1.18	0.22
Translation *x*	19.23	2.98	1.59	0.27	1.16	0.18
Translation *y*	19.51	4.16	1.59	0.37	1.17	0.25
Translation *z*	18.61	3.93	1.56	0.34	1.12	0.23
Rotation *x*	29.08	20.63	2.40	1.75	1.81	1.29
Rotation *y*	25.94	14.88	2.14	1.26	1.60	0.92
Rotation *z*	19.10	2.71	1.59	0.23	1.15	0.16
Translation	19.05	4.12	1.56	0.35	1.14	0.24
Rotation	25.55	14.97	2.11	1.27	1.57	0.93
Rotation and translation	26.24	15.66	2.14	1.31	1.60	0.96

*CO* camera obcura,* FCM* full camera model.

The remaining rows in Table 3 state the results for the full camera and camera obscura models under specific conditions. The second row indicates the errors for translation in specific directions; translation generally produced an error of approximately 4 pixels with the full camera model and approximately 19 pixels with the camera obscura model. Regarding rotation, the full camera model behaved differently in *z*-axis rotation than rotation on the other axes. *z*-axis rotation produced a very low error of 2.71 pixels, while rotation on the *x* and *y* axes produced an absolute error of approximately 25 pixels. The causes of this behaviour are discussed in Section 6. The last row of results in the table shows errors for translation, rotation and concurrent rotation and translation. The results for these data sets correspond to the previously mentioned properties. The translation error produced by the full camera model (4.12 pixels) is less than error produced by the camera obscura model. The errors for rotation and concurrent translation and rotation time are greater than the errors for translation error, however the full camera model still produced better results than the camera obscura model.

**Figure 11:**
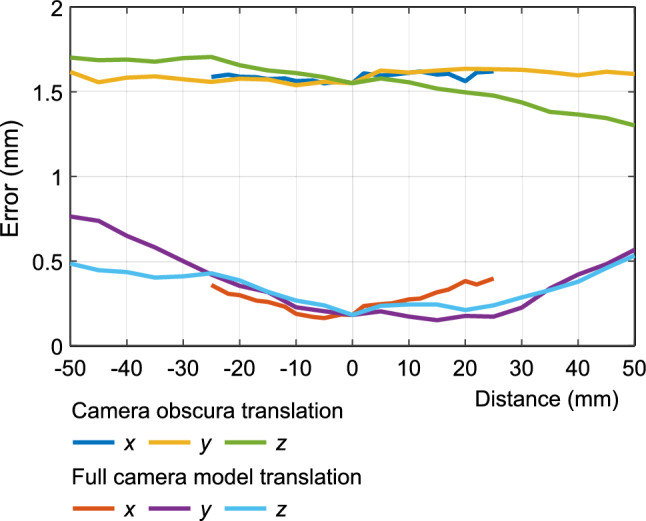
Comparison of absolute error in millimeters produced by the camera obscura and full camera models for various input data sets.

Figure 11 graphically compares the average absolute errors of the two camera models, clearly indicating a lower absolute error (in pixels) produced by the full camera model in every case.

**Figure 12:**
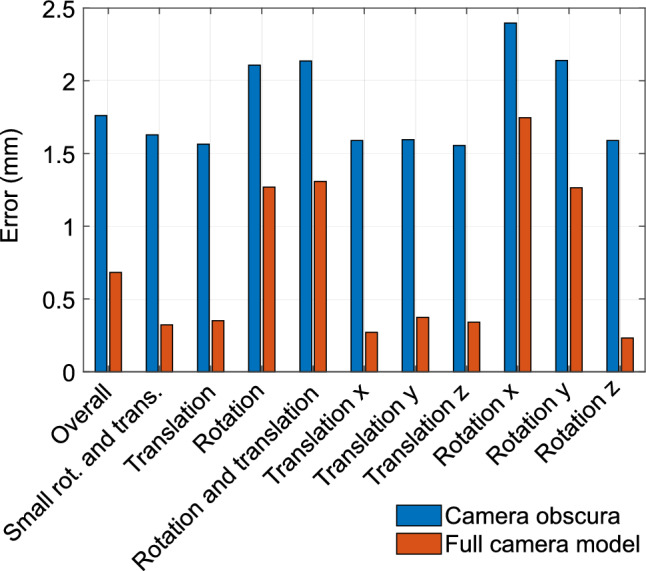
Comparison of absolute error in millimeters for translation o then *x*, *y* and *z* axes.

Figure 12 graphs the absolute errors in pixels for translation, indicating that the absolute error was lower with the full camera model than the camera obscura model. It is clear that the error produced by the camera obscura model is largely independent of translation, while full camera model produced greater absolute errors for larger translations and a lower errors for smaller translations.

**Figure 13:**
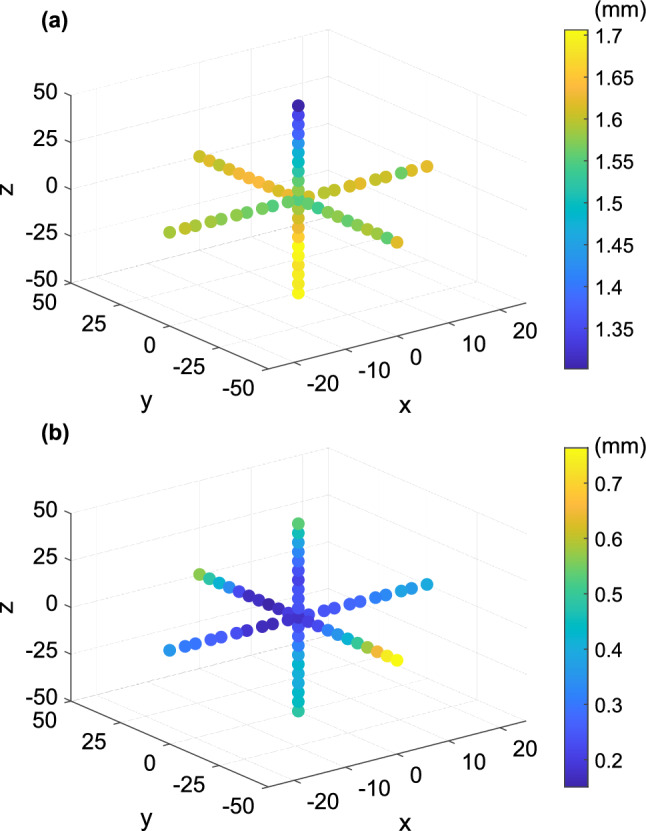
Graphical representation of absolute error in millimeters in three dimensions for translation: (**a**) camera obscura; (**b**) full camera model.

Figure 13 plots the absolute errors in pixels for translation in three dimensions. Figure 13a and b respectively indicate the absolute errors produced by the camera obscura model and the full camera model. The absolute errors on the *x* and *y* axes are roughly symmetrical, indicating that the errors produced are not dependent on the direction of movement left or right. The absolute error on the *z* axis is asymmetrical, becoming lower as the camera moved nearer to the pattern (the image of the calibration pattern is larger) and larger when the camera moved away (the image of the calibration pattern is smaller). It is also clear that with the full camera model, the points with smallest absolute error are concentrated near the base point, i.e. very small distances from the zero position.

**Figure 14:**
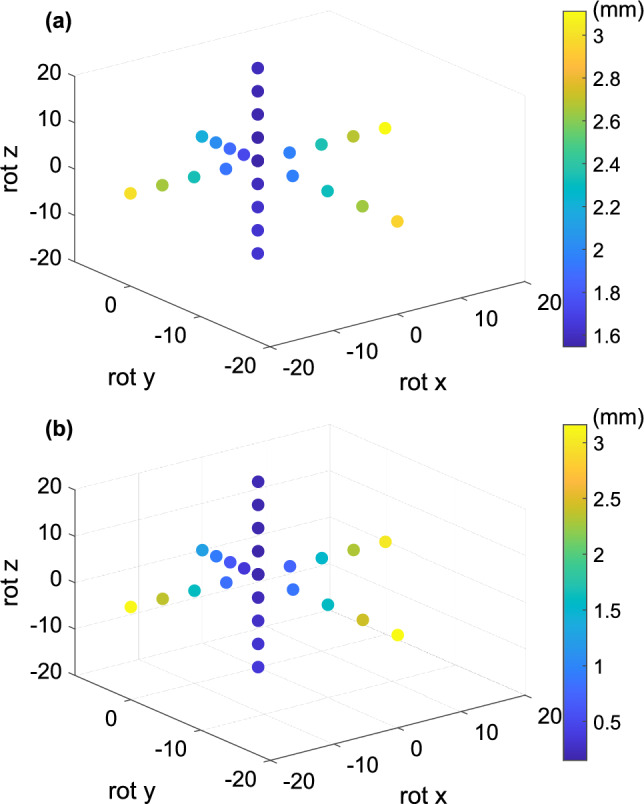
Graphical representation of absolute error in millimeters in three dimensions for rotation: (**a**) camera obscura; (**b**) full camera model.

Figure 14 plots the absolute errors in pixels for rotations in three dimensions. Figure 14a and b respectively indicate the absolute errors produced by the camera obscura model and the full camera model. Both camera models produced a similar distribution of absolute errors, but the full camera model’s absolute error was lower. Both algorithms produced the lowest absolute error in *z*-axis rotation. In this case, the calibration pattern image was affected by rotational transformation only, whereas *x* and *y* axis rotation affected the calibration pattern image with projective transformation, and the resulting absolute error was greater.

## Discussion

The study raises several discussion questions. The first question concerns the accuracy of the presented results.The full camera model was compared with the widely recognized camera obscura model, and an input data set was obtained experimentally from a real robot and camera equipment. To obtain the most accurate results as possible, the robotic arm was calibrated with an an advanced procedure. The total error in the input data set was estimated at 0.14 mm. The estimated data set error was less than the resulting errors produced by the camera obscura and full camera models, therefore the input data set was suitable as a reference data set to compare the camera models.

The next question concerns the applicability of the proposed full camera model. The study used a test robot stand and 100 mm calibration pattern. Pilot testing indicated an average absolute error of 0.3 mm to 0.7 mm. The absolute error in pixels is generally independent of the camera type and calibration pattern size. If we consider a calibration pattern ten times smaller used in combination with telephoto lens, the absolute error will also fall in the range of 0.03 mm to 0.07 mm, which is sufficient for current robotic manipulators.

The final question for discussion concerns the inaccuracy encountered in rotation on the *x* and *y* axes. The full camera model expects the TCP to be in the center of the lens. The position of the sensing element was also required, but unfortunately, the camera manufacturers do not specify the parameter *L* , and it therefore had to be determined experimentally. To conduct a sensitivity analysis of the parameter *L*, we estimated the derivative of the absolute error (in millimeters) with respect to *L* using the finite difference method, as detailed in Equation (23). Our numerical experiments revealed that at $L=9.5$ mm, the computed average derivative is approximately 0.0024 mm. This finding suggests that the selected value of *L* is near-optimal. Deviations from $L=9.5$ mm resulted in a marked increase in the magnitude of the derivative, indicating heightened sensitivity to changes in *L*.

An important consideration of the proposed solution is computational cost. Both algorithms were executed in Matlab software on a Dell Inspiron 7306 2n1 computer with a 2.4 GHz i5-1135G7 processor, which has a computational power similar to the target platform, which is an industrial computer. The entire calculation for the full camera model is a long mathematical expression which could be easily optimized (and parallelized) and implemented in another programming language such as C++, C# or a specific robot programming language. Table 4 compares the computational costs of the camera obscura and full camera models. The computational cost of the full camera model is significantly higher than camera obscura model. Although the calibration procedure is iterative, the full camera model requires only approximately 100–1000 iterations. The full camera model is therefore suitable for industrial processes because an industrial computer is able to process millions of numerical operations in hundreds of milliseconds, and especially because it is expected that a robot is calibrated at least once per day, usually after warming up.

**Table 4:** Summary of the computational costs for the camera obscura and full camera models.

	Sin	Cos	Mult.	Div.	Add.	Sub.
CA	3	3	83	20	42	17
FCM	3	3	1.3 ths	20	1.5 ths	1.2 ths

*CA * camera obscura,* FCM* full camera model,* Mult.* multiplication,* Div.* division,* Add.* addition,* Sub.* subtraction.

Table 5 summarizes the overall characteristics of the camera models. A comparison of the full camera model with the camera obscura model clearly indicates its major advantage in greater precision. Implementation of both models is simple. The full camera model requires the calculation of a lengthy, single mathematical expression, but this could be easily optimized. The camera obscura model involves calculation of a sequence of mathematical expressions. The computational costs for the full camera model are higher than the camera obscura model, however they are acceptable for both. The full camera model demonstrates two more advantages in variable image distance and variable focal length. The camera obscura has a fixed image distance, and focal length is considered.

**Table 5:** Summary of overall characteristics.

	Full camera model	Camera obscura
Precision	High	Low
Implementation	Easy	Easy
Computational costs	Low	Very low
Image distance	Variable	Fixed
Focal length	Variable	Not applicable

## Conclusion

The study proposed a full mathematical model of an industrial camera and measured a reference data set with an experiment for comparison to a generally known camera obscura model.

The results of the experiment indicated that the proposed mathematical model of a full camera produced a significantly lower absolute error than the camera obscura model. The absolute error was approximately 2.65 times lower, and for small translations and rotations, up to 5.38 times lower. The novelty of the proposed solution was discussed in relation to other state-of-the-art methods.

Future studies will test the hypothesis that the proposed mathematical full camera model is able to obtain a lower absolute error with smaller calibration patterns and a telephoto lens. Other potential work is the application of the full camera model to provide precise calibration in a real industrial application.

## Data Availability

The data supporting the findings of this study are available upon request. These data have been collected and are accessible for further analysis and validation. Please contact Jaromir Konecny at jaromir.konecny@vsb.cz to request access to the data.
